# Association of *Salmonella* Serotypes with Quinolone Resistance in Broilers

**DOI:** 10.14252/foodsafetyfscj.2018012

**Published:** 2018-12-21

**Authors:** Ayumi Nakatsuchi, Mizuho Inagaki, Michiyo Sugiyama, Masaru Usui, Tetsuo Asai

**Affiliations:** 1 Faculty of Applied Biological Science, Gifu University, 1-1 Yanadigo, Gifu 501-1193, Japan; 2 United Graduate School of Veterinary Science, Gifu University, 1-1 Yanadigo, Gifu 501-1193, Japan; 3 School of Veterinary Medicine, Rakuno Gakuen University, Ebetsu, Hokkaido 069-8501, Japan

**Keywords:** broiler chicken, fluoroquinolone, quinolone resistance, *Salmonella*

## Abstract

Fluoroquinolone is widely used for the treatment of bacterial diseases, and the emergence of quinolone resistance has become a serious concern in recent years, owing to an increase and inappropriate use of antimicrobials. Here, we attempted to understand the differences in the emergence frequency of quinolone-resistant bacterial variants in three *Salmonella* serotypes *S*. Infantis, *S*. Schwarzengrund, and *S*. Manhattan—which are mainly found in broiler industries in Japan. Emergence frequency tests for quinolone-resistant variants using enrofloxacin-containing agar plates and sequence analysis in the quinolone resistance-determining region (QRDR) of *gyrA* in DNA gyrase were performed. The results showed no significant difference in the emergence frequency among the three serotypes, and most of the resistant variants had mutations in the QRDR region. These findings suggest that differences in the serotypes tested are not associated with the emergence frequency of quinolone-resistant variants.

## Introduction

Fluoroquinolone is a widely used antimicrobial agent for the treatment of bacterial infections in humans. It is used as a first-line treatment for severe gastroenteritis, including *Salmonella* infection, in adults^1^^)^. Emergence of fluoroquinolone-resistant *Salmonella* increases the risk of treatment failure in patients. At present, three fluoroquinolones—norfloxacin (NFLX), ofloxacin (OFLX), and enrofloxacin (ERFX)—have been approved for the treatment of bacterial diseases in broiler chicken in Japan. To date, although fluoroquinolone resistance has been reported in limited serotypes (*S.* Typhimurium in cattle and *S.* Chorelaesuis in pigs) of *Salmonella* in Japan^2^^,^^3^^)^, fluoroquinolone resistance has not been observed in *Salmonella* isolates from poultry.

The development of fluoroquinolone resistance in *Enterobacteriaceae,* including *Salmonella,* can be associated with multiple substitutions of amino acids in the target enzymes (DNA gyrase and topoisomerase IV), decreased permeability of drugs, and/or activation of efflux mechanisms^4^^)^. A point mutation in the quinolone resistance-determining region (QRDR) of *gyrA*, which encodes the GyrA subunit of DNA gyrase, has been recognized to be responsible for quinolone resistance^5^^)^. Moreover, additional mutations in the QRDR of *gyrA* and *parC* are required for resistance development to fluoroquinolones^4^^)^. The emergence of quinolone-resistant *Salmonella* with a QRDR mutation has been associated with efflux pump activation in experimental investigations^6^^)^. Additionally, activation of the AcrAB–TolC efflux pump has been reported to be associated with the emergence of quinolone resistance in *S.* Typhimurium^7^^)^ and *S.* Choleraesuis^8^^)^. Therefore, evaluation of emergence frequencies of quinolone-resistant strains and efflux pump activities of the strains may contribute to the estimation of fluoroquinolone-resistance development potential in the strains.

*Salmonella* causes various foodborne illnesses and is transferred from animals to humans via animal products^1^^)^. Chicken meat is a common source of foodborne salmonellosis in Japan^9^^)^. *Salmonella enterica* subsp. *enterica* serovar Infantis is known to be the major serotype present in broiler meat in Japan^10^^)^. In the last decade, not only *S.* Infantis but also *S.* Schwarzengrund and *S.* Manhattan have been isolated from broiler samples^11^^)^. Of the three serotypes prevalent in the Japanese broiler industry, fluoroquinolone-resistant *S.* Schwarzengrund has been reported in Thailand and Taiwan as well^12^^,^^13^^)^. As a change in *Salmonella* serotypes prevalent in broiler was observed in Japan, we aimed to determine the possibility of quinolone resistance emergence among three *Salmonella* serotypes. Therefore, in this study, we investigated the frequency of emergence of quinolone-resistant mutants and their efflux pump activities using *S.* Infantis, *S.* Schwarzengrund, and *S.* Manhattan isolates from broiler chickens.

## Materials and Methods

A total of 48 nalidixic acid (NA)-susceptible *Salmonella* isolates, including 14 strains of *S*. Infantis, 16 strains of *S*. Schwarzengrund, and 18 strains of *S*. Manhattan, from broiler chickens and retail chicken meats collected between 2010 and 2013, were used. The minimum inhibitory concentrations (MICs) of NA and ERFX were determined using broth microdilution methods with commercially available plates (Eiken Chemical Co., Ltd. Tokyo, Japan).

The emergence frequency of quinolone-resistant variants was determined as the ratio of the average number of colonies on agar plates with and without fluoroquinolone. Each strain suspension was adjusted to 10^10^ CFUs/mL and inoculated onto Mueller Hinton (MH) agar plates containing different concentrations of ERFX (4 × MIC (4MIC) and 2 × MIC (2MIC)).

Mutation in the QRDR domain of *gyr*A was analyzed by direct DNA sequencing using two selected strains that appeared in each of the fluoroquinolone-containing agar plates. Briefly, to extract bacterial DNA, the bacterial suspension was boiled in distilled water and centrifuged at 10,000 *g* for 5 min. The supernatant was stored at -20°C as template DNA. Next, *gyr*A was amplified using TaKaRa ExTaq (TaKaRa Bio Inc., Kusatsu, Japan) with previously reported primer sets (STGYRA1 and STGYRA2)^14^^)^. The PCR conditions were as follows: 94°C for 3 min, 35 cycles at 94°C for 30 sec, 55°C and 72°C for 30 sec, and 72°C for 10 min. The amplified PCR product was purified using Wizard^®^ SV Gel and PCR Clean-UP System (Promega, Fitchburg, WI, USA) according to the manufacturer’s protocol. DNA sequencing was performed on an ABI Prism 3130 Genetic Analyzer using a BigDye Terminator ver. 3.1 Cycle Sequencing Kit (Thermo Fisher Scientific, Waltham, MA, USA).

Quantitative PCR analysis of AcrB expression was performed as described by Usui et al^15^^)^. Briefly, bacterial RNA was extracted using ISOGEN (Nippon Genetics Co. Ltd., Tokyo, Japan). To eliminate genomic DNA, the extracted RNA was treated with recombinant DNase I (TaKaRa Bio Inc.). cDNA was synthesized from these RNA samples using a PrimeScript RT reagent kit (TaKaRa Bio Inc.). Real-time PCR was performed using the Step One Plus^TM^ Real-Time PCR system (Thermo Fisher Scientific), gene-specific primers^7^^,^^16^^)^ (*acrB*: acrB-rt1 and acrB-rt2; *16S rRNA*: Salm 16S-F and Salm 16S-R1), and the THUNDERBIRD^®^ SYBR qPCR Mix (Toyobo Co., Ltd, Osaka, Japan). The PCR conditions were as follows: 95°C for 1 min, followed by 40 cycles at 95 °C for 10 s, 60°C for 15 s, and 72°C for 30 s. The expression of *acrB* was normalized with respect to that of *16S rRNA*. The ΔΔ*Ct* method was used to calculate fold induction of transcription of a target gene by comparison with a value relative to wild-type strain growth in MH broth.

All statistical analyses to determine differences were performed using one-way analysis of variance followed by Tukey’s multiple comparison test. *P*-values < 0.05 indicated significance.

## Results and Discussion

**Fig. 1.**
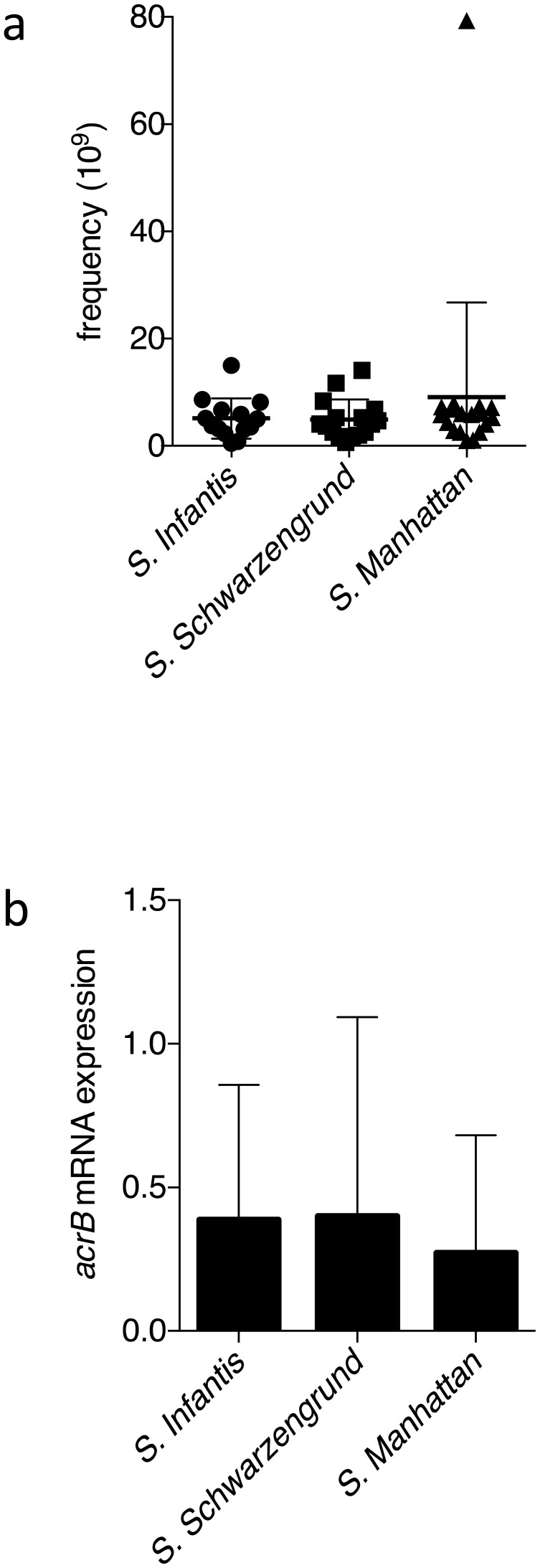
Emergence frequency of quinolone-resistant variants (a) and *acrB* mRNA expression level of the quinolone-resistant mutants (b) among the three *Salmonella* serotypes.

The resistant variants of all three serotypes did not emerge on MH agar containing 4MIC of ERFX. Following culture on MH agar containing 2MIC of ERFX, the average emergence frequencies of the resistant variants were 5.1×10^−9^, 5.0×10^−9^, and 9.1×10^−9^ for *S.* Infantis (n = 14), *S*. Schwarzengrund (n = 16), and *S*. Manhattan (n = 18), respectively (Fig. 1a). No significant difference in the emergence frequency of quinolone-resistant variants was observed. To determine mutations in the QRDR region of DNA gyrase, two variants per parental strain were selected and subjected to direct DNA sequencing. In the selected variants, the Ser-83 or Asp-87 mutations in *gyrA* were found in 85 of the 96 variants (Table 1). The Ser-83/Asp-87 mutation rates were 42.8/57.1, 50.0/46.9, and 41.7/30.6% in *S.* Infantis, *S*. Schwarzengrund, and *S*. Manhattan, respectively, indicating that the Ser-83/Asp-87 mutation is observed in most of the resistant strains of the three serotypes. A previous study in Japan showed a higher prevalence of NA resistance in *S*. Schwarzengrund (21.4%) than in *S.* Infantis (8.0%) and *S*. Manhattan (11.8%), although fluoroquinolone resistance was not observed in these strains^11^^)^.

**Table 1.** MICs and *GyrA* sequencing data of variants isolated from the parental strain of three *Salmonella* serotypes

GyrA		*S. *Infantis			*S.* Schwarzengrund			*S. *Manhattan		
Amino acid substitution		n	NA MIC	ERFX MIC	n	NA MIC	ERFX MIC	n	NA MIC	ERFX MIC
Ser-83	TTC	10	512->512	0.03-1	10	>512	1-2	11	512->512	0.5-1
	TAC	2	>512	1	6	>512	0.5-1	4	512->512	1
	Subtotal	12			16			15		
Asp-87	GGC	9	256-512	0.5-1	7	256->512	0.5-1	2	128-256	0.5
	TAC	5	256-512	0.5	4	512->512	0.5-1	7	512->512	0.5-2
	AAC	2	512->512	0.5-1	4	512->512	0.5-1	1	512	1
	CAC							1	512	1
	Subtotal	16			15			11		
Wildtype		0			1	256	0.5	10	16-256	0.5-2
Total		28			32			36		

Next, to evaluate the efflux pump activities, we examined the expression levels of *acrB* in the three serotypes. The average *acrB* expression levels in *S.* Infantis (n = 14), *S*. Schwarzengrund (n = 16), and *S*. Manhattan (n = 18) were 0.39-, 0.40-, and 0.27-fold of the expression in *S*. Infantis ATCC 51741, respectively (Fig. 1b). Thus, no significant differences were observed in the average expression of *acrB* in the three serotypes. Our previous study showed higher *arcB* expression in quinolone-resistant *Salmonella* strains than in quinolone-susceptible strains^8^^)^. In this study, the susceptible strains were selected to evaluate the potential emergence of quinolone resistance in each serotype of *Salmonella.* Additionally, following antimicrobial drug treatment, the *Salmonella* strains showed increased activation of the AcrAB–TolC efflux pump^15^^,^^17^^)^. Further, as *Salmonella* strains isolated from poultry samples were used, it is unknown whether the bacteria were previously exposed to antimicrobials.

The fluoroquinolone-resistant *Salmonella* serotype has been reported worldwide^2^^,^^3^^,^^12^^,^^13^^,^^18^^)^. However, the fluoroquinolone-resistant strain of *S*. Infantis found in Serbia exhibited high clonality^18^^)^. Moreover, in Japan, fluoroquinolone-resistant strains of *Salmonella* in food-producing animals rarely emerged^2^^,^^3^^)^. Although the frequency of emergence of quinolone resistance among the three *Salmonella* serotypes was not different in this study, continuous surveillance for antimicrobial susceptibility in *Salmonella* from food-producing animals is essential to prevent the spread of the resistant bacteria imposed by novel risk factors.

## References

[1] Acheson D, Hohmann EL. Nontyphoidal Salmonellosis. Clin Infect Dis. 2001; 32: 263–269. 10.1086/318457 11170916

[2] Esaki H, Chiu CH, Kojima A, et al. Comparison of fluoroquinolone resistance genes of *Salmonella enterica* serovar Choleraesuis isolates in Japan and Taiwan. Jpn. J. Infect. Dis. 2004; 57: 287–288. 15623959

[3] Kawagoe K, Mine H, Asai T, et al. Changes of multi-drug resistance pattern in *Salmonella enterica* subspecies enterica serovar typhimurium isolates from food-producing animals in Japan. J Vet Med Sci. 2007; 69: 1211–1213. 10.1292/jvms.69.1211 18057843

[4] Piddock LJV. Fluoroquinolone resistance in *Salmonella* serovars isolated from humans and food animals. FEMS Microbiol Rev. 2002; 26: 3–16. 10.1111/j.1574-6976.2002.tb00596.x 12007640

[5] Griggs DJ, Gensberg K, Piddock LJ. Mutations in *gyrA* gene of quinolone-resistant *Salmonella* serotypes isolated from humans and animals. Antimicrob Agents Chemother. 1996; 40: 1009–1013. 10.1128/AAC.40.4.1009 8849216 PMC163248

[6] Ricci V, Tzakas P, Buckley A, Piddock LJV. Ciprofloxacin-resistant *Salmonella enterica* serovar Typhimurium strains are difficult to select in the absence of AcrB and TolC. Antimicrob Agents Chemother. 2006; 50: 38–42. 10.1128/AAC.50.1.38-42.2006 16377664 PMC1346778

[7] Zheng J, Cui S, Meng J. Effect of transcriptional activators RamA and SoxS on expression of multidrug efflux pumps AcrAB and AcrEF in fluoroquinolone-resistant *Salmonella* Typhimurium. J Antimicrob Chemother. 2009; 63: 95–102. 10.1093/jac/dkn448 18984645

[8] Usui M, Uchiyama M, Iwanaka M, Nagai H, Yamamoto Y, Asai T. Intracellular concentrations of enrofloxacin in quinolone-resistant *Salmonella enterica *subspecies* enterica *serovar Choleraesuis. Int J Antimicrob Agents. 2009; 34: 592–595. 10.1016/j.ijantimicag.2009.07.009 19733466

[9] Noda T, Murakami K, Ishiguro Y, Asai T. Chicken meat is an infection source of *Salmonella* serovar Infantis for humans in Japan. Foodborne Pathog Dis. 2010; 7: 727–735. 10.1089/fpd.2009.0438 20141347

[10] Asai T, Ishihara K, Harada K, et al. Long-term prevalence of antimicrobial-resistant *Salmonella enterica* subspecies *enterica* Serovar infantis in the broiler chicken industry in Japan. Microbiol Immunol. 2007; 51: 111–115. 10.1111/j.1348-0421.2007.tb03881.x 17237606

[11] Sasaki Y, Ikeda A, Ishikawa K, et al. Prevalence and antimicrobial susceptibility of *Salmonella* in Japanese broiler flocks. Epidemiol Infect. 2012; 140: 2074–2081. 10.1017/S0950268812000039 22281015

[12] Akiyama T, Khan AA. Molecular characterization of strains of fluoroquinolone-resistant *Salmonella enterica* serovar Schwarzengrund carrying multidrug resistance isolated from imported foods. J Antimicrob Chemother. 2012; 67: 101–110. 10.1093/jac/dkr414 22010209

[13] Baucheron S, Chaslus-Dancla E, Cloeckaert A, Chiu CH, Butaye P. High-level resistance to fluoroquinolones linked to mutations in *gyrA, parC*, and *parE* in *Salmonella enterica* serovar Schwarzengrund isolates from humans in Taiwan. Antimicrob Agents Chemother. 2005; 49: 862–863. 10.1128/AAC.49.2.862-863.2005 15673791 PMC547372

[14] Giraud E, Brisabois A, Martel JL, Chaslus-Dancla E. Comparative studies of mutations in animal isolates and experimental in vitro- and in vivo-selected mutants of *Salmonella* spp. suggest a counterselection of highly fluoroquinolone-resistant strains in the field. Antimicrob Agents Chemother. 1999; 43: 2131–2137. 10.1128/AAC.43.9.2131 10471553 PMC89435

[15] Usui M, Nagai H, Hiki M, Tamura Y, Asai T. Effect of antimicrobial exposure on AcrAB expression in *Salmonella enterica* subspecies *enterica* serovar Choleraesuis. Front Microbiol. 2013; 4: 53. 10.3389/fmicb.2013.00053 23503095 PMC3596762

[16] Fey A, Eichler S, Flavier S, Christen R, Höfle MG, Guzmán CA. Establishment of a real-time PCR-based approach for accurate quantification of bacterial RNA targets in water, using *Salmonella* as a model organism. Appl Environ Microbiol. 2004; 70: 3618–3623. 10.1128/AEM.70.6.3618-3623.2004 15184165 PMC427797

[17] Usui M, Uchiyama M, Baba K, Nagai H, Yamamoto Y, Asai T. Contribution of enhanced efflux to reduced susceptibility of *Salmonella enterica* serovar Choleraesuis to fluoroquinolone and other antimicrobials. J Vet Med Sci. 2011; 73: 279–282. 10.1292/jvms.10-0309 20953129

[18] Velhner M, Kozoderović G, Grego E, et al. Clonal spread of *Salmonella enterica* serovar Infantis in Serbia: acquisition of mutations in the topoisomerase genes gyrA and parC leads to increased resistance to fluoroquinolones. Zoonoses Public Health. 2014; 61: 364–370. 10.1111/zph.12081 24119387

